# Determining the linear correlation between dielectric spectroscopy and viable biomass concentration in filamentous fungal fermentations

**DOI:** 10.1007/s10529-023-03384-w

**Published:** 2023-05-25

**Authors:** Atli Magnússon, Jari Pajander, Gürkan Sin, Stuart Stocks

**Affiliations:** 1 LEO Pharma A/S, Ballerup, Denmark; 2 Department of Chemical and Biochemical Engineering, Technical University of Denmark, Lyngby, Denmark

**Keywords:** Dielectric spectroscopy, Fermentation, Sensor calibration, Viable biomass

## Abstract

**Objectives:**

Dielectric spectroscopy is commonly used for *online* monitoring of biomass growth. It is however not utilized for biomass concentration measurements due to poor correlation with Cell Dry Weight (CDW). A calibration methodology is developed that can directly measure viable biomass concentration in a commercial filamentous process using dielectric values, without recourse to independent and challenging viability determinations.

**Results:**

The methodology is applied to samples from the industrial scale fermentation of a filamentous fungus, *Acremonium fusidioides*. By mixing fresh and heat-killed samples, linear responses were verified and sample viability could be fitted with the dielectric $\Delta C$ values and total solids concentration. The study included a total of 26 samples across 21 different cultivations, with a legacy at-line viable cell analyzer requiring 2 ml samples, and a modern on-line probe operated at-line with 2 different sample presentation volumes, one compatible with the legacy analyzer, a larger sample volume of 100 ml being compatible with calibration for on-line operation. The linear model provided an ${R}^{2}$ value of 0.99 between $\Delta C$ and viable biomass across the sample set using either instrument. The difference in ∆C when analyzing 100 mL and 2 mL samples with an in-line probe can be adjusted by a scalar factor of 1.33 within the microbial system used in this study, preserving the linear relation with ${R}^{2}$ of 0.97.

**Conclusions:**

It is possible to directly estimate viable biomass concentrations utilizing dielectric spectroscopy without recourse to extensive and difficult to execute independent viability studies. The same method can be applied to calibrate different instruments to measure viable biomass concentration. Small sample volumes are appropriate as long as the sample volumes are kept consistent.

## Introduction

The concentration of viable biomass is a key process parameter for any bioprocess. Despite its importance, it is extremely difficult and time-consuming to measure (Flores-Cosío et al. 2020), and for some organisms, it is practically impossible. Even for simple organisms, measuring active or viable biomass is subjective and may not be practical (Wang et al. 2021). The most widely accepted standard technique for quick estimations of biomass is the use of Optical Density calibrated to Cell Dry Weights (CDW) (Madrid and Felice 2005). However, these techniques measure total biomass, including dead cells which may lead to inaccuracies when determining strain parameters like observed yield. Furthermore, CDW can not differentiate between biomass and non-biological solids present in the media.

Dielectric spectroscopy is the only known method that allows for *online* monitoring of viable biomass (Yardley et al. 2000, Flores-Cosío et al. 2020); In part of the radio-frequency range (for one of the instruments in this study, 64 to 20000 kHz), permittivity is dominated by the capacitive behaviour of cell membranes of intact cells. The frequency-dependence of the media permittivity in this region is also known as the β-dispersion. Bio-capacitance is widely used in the fermentation and cell culture industries for monitoring purposes. The trends can be utilized to detect abnormalities in cellular growth (Patel and Markx 2008) or even directly adapted for developing control strategies (Ehgartner et al. 2017). However, there is value in being able to monitor viable biomass concentrations instead of dielectric properties. Biomass concentration is widely utilized for detailed process modelling, control, establishing growth kinetics, yield, and stoichiometry which are critical in process optimization and intensification (Fernandes et al. 2013). Unfortunately, there is presently no universal relation between the results of dielectric spectroscopy and active biomass concentration that is transferable between different biological systems. Most efforts to calibrate *online* measurements to biomass rely on establishing a correlation between permittivity increments and filterd or centrifuged CDW. This initially works well for systems starting with initially low biomass concentration, but the correlation tends to break down as the process progresses and biomass concentrations increase (Krairak et al. 1999; Rønnest et al. 2011). While sometimes said to be correlated to a morphological change, another obvious cause is the accumulation of non-viable biomass, cell debris or other non-soluble solids during the fermentation period, which continue to contribute to the filtered mass, but not the permittivity signal (Krairak et al. 1999; Maskow et al. 2008).

The probes measure “viable biomass”. The term viable biomass is not clearly defined, and different viability measurement techniques give different results depending on what property it is they specifically measure (Kell et al. 1998, Flores-Cosío et al. 2020). Dielectric spectroscopy only measures changes in dielectric properties which are usually directly influenced by the amount of cytoplasm surrounded by an intact plasma membrane and thus indirectly estimates membrane integrity (Patel and Markx 2008). It has been established for unicellular organisms, like mammalian cells or yeast, that dielectric spectroscopy can measure viable cell densities regardless of different cell growth phases, where for these relatively large unicellular organisms, automated independent methods for viability estimations can be applied (Lee et al. 2015; Flores-Cosío et al. 2019). Dielectric spectroscopy is not limited to these types of organisms so a similar correlation between viable biomass and dielectric spectroscopy should exist for most or all bioprocesses. The viability of a filamentous organism is extremely difficult to measure, CFU’s (Colony Forming Units) being completely meaningless, the only reliable protocols rely on fluorescent staining and quantitative image analysis which are expensive and time-consuming to develop (e.g. Stocks 2004; Véronique et al. 2007), and out of reach for many researchers. A quick and easy estimation of active biomass concentration for these types of organisms would be invaluable for those interested in going beyond analyzing data trends and attempting to develop e.g. a process model.

In this work, dielectric spectroscopy was applied to samples of an industrial filamentous fungal fermentation. An elegant calibration method where fresh and heat-killed sample is mixed in controlled proportions to verify linearity of the dielectric response is presented, then an expanded collection of samples then allows calibration to viable biomass concentration through a fitting exercise. The purpose of the calibration methodology is to discover the underlying correlation between viable biomass and dielectric spectroscopy. This approach is not dependent on any time consuming independent method for viability estimation, and may be of use to others working in the field, where access to the resources for such independent methods are rare.

## Methods

### Microorganism and media conditions

Samples were obtained from the large-scale stainless steel main bioreactors used for commercial manufacturing of Fusidic Acid at the Ballerup site of LEO Pharma A/S. Fresh samples were taken from these industrial fermentations from various times of cultivation. The conditions and media are similar to the process description of Fusidic Acid fermentation reported by Daehne (1984) using *Acremonium fusidioides,* exact details regarding present-day operating conditions, component concentrations, and organism are considered sensitive information and are not disclosed.

### Cell dry weight

Cell Dry Weight (CDW) measurements are performed by passing a known mass of fermentation broth through a 70 mm glass fiber filter paper with an applied vacuum. The filtrate is washed two times by filling the funnel with deionized water. The washed filtrate was then placed in a drying oven at a temperature of 100 °C for at least 48 h. Final CDW values are expressed in unit dry weight per unit fermentation broth weight.

### Dielectric spectroscopy

Legacy analyzer (ABER Viable Cell Analyzer Model 822): This device can only be used at-line, permittivity is measured at a single frequency of 1 kHz in a built-in sample chamber stirrer that can take up to 2 mL of a sample along with a stirrer and temperature control. To measure ∆C a small portion of the fresh sample was filtered and the permittivity of the cell-free filtrate is recorded and utilized as the media background.

Contemporary analyzer (ABER Futara pico + amplifier, parts 5310–00 + 5320–00): The probe was operated at-line with the default microbial settings, a dual-frequency measuring pF/cm at 580 kHz and 15,650 kHz, the difference reported as ∆C. In this case the manufacturer recommends a sample container of at least 8 cm diameter to enable establishment of a suitable electric field; 100 ml samples were presented in 10 cm square 250 ml PETG constainers (Nalgene™ part nr. 2019–0250). However, when comparing to the legacy analyzer or operating very small bioreactors, it is often necessary to constrain sample volumes as much as possible, so 2 ml samples (as per-legacy analyzer) were also presented, in 12 ml polystyrene tubes (Nunc™ part nr. 342,919).

Samples: A total of 26 samples were obtained from 21 independent production scale cultivations. An enabling feature of the calibration procedure presented later, is the mixing of heat killed and fresh sample: A portion of each sample was moved to a separate container and heat-killed by placing the container in a water bath for 30 min at 70 °C. Heat killed and fresh samples were mixed at approximately 0%, 25%, 50%, 75%, and 100% w/w, with the actual proportions being accurately weighed for the purpose of the later calibration (so called “viability control” Véronique et al. 2007). Of the 26 samples, 15 samples were analyzed using the Futara pico probe. The rest of the samples were analyzed using the legacy viable cell analyzer. This is to address any systematic differences that may occur in dielectric spectroscopy readings because of the different measurement techniques employed.

### Calibration procedures

Using the described linear relationships, the calibration methodology exploits the added presence of the mixed fresh/killed samples in finding the parameters of the relationship in Eqs. 1 and 2. ∆C is the result from the measurement (pF/cm), *X*_viable_ is the viable biomass concentration (g/kg), β_1_ & β_2_ are constants that assure a linear relationship.

1 $$\Delta C={\upbeta }_{1}{X}_{Viable}+{\upbeta }_{2}$$

β_2_ is, in principle, easy to estimate from killed samples. Estimating β_1_ is a much more challenging task as *X*_viable_, in the original fresh sample, is not known. However, *X*_viable_, can be written as a relationship to CDW (${X}_{Dry}$) as

2 $${X}_{Viable}=\mathrm{\alpha }{M}_{Frac}{X}_{Dry}$$

where $\mathrm{\alpha }$ denotes the viable fraction of the filtered dry weight of the fresh sample, which can take a value from 0 to 1 and is unique for each fresh sample. Here we have also introduced the mass fraction of the fresh sample *M*_Frac_, the expected linear relationship between ∆C and the mixture ratio was checked for any given fresh sample (examples will be presented). Then, by including all the data collected from fresh, mixed and killed samples, in an optimization/fitting exercise the values for the constants and the initial viability for each fresh sample can be solved; the optimization is formulated as follows:

3 $${\mathrm{\alpha }}_{opt}=\underset{\mathrm{\alpha }}{\mathrm{argmax}}\space{R}^{2}$$

$$0 \le \alpha \le 1$$

This was first solved with MATLAB’s *fmincon()* function which utilizes an Interior Point algorithm, searching for β_1_, β_2_ and values for α (MATLAB R2021a). The task was surprisingly trivial and was later easily demonstrated in Microsoft Excel using the native *GRG Nonlinear* solver to maximize R^2^ (Office 365, Version 2202, build 14,931.20806). This can be used more generally (see supplementary material “Excel file” for an example). Note that the data presented in this work have been arbitrarily scaled to preserve industry-sensitive information and thus exact units will not be included on the axes. Axis ticks are preserved to illustrate the overall trend.

## Results and discussion

### Relationship between dielectric spectroscopy and active biomass

Some trivial controls are shown in the supplementary material: Measurements of a sample from a bioreactor on the viable cell analyser show no significant change in the permittivity signal even after leaving in an open container for 90 min at ambient conditions, indicating that this microbial system is stable throughout the analysis. Also, subjecting broth to heat treatment by placing the sample container in a water bath at 70 °C led to rapid loss of permittivity signal, the signal stabilized at low permittivity measurements after 15 min, (Supplementary Fig. A.1). Some residual broth permittivity remains even after further heat treatment, but the microbes are considered dead as permittivity does not decrease further with longer heat treatment. Heat treated samples are considered non-viable.

**Fig. 1:**
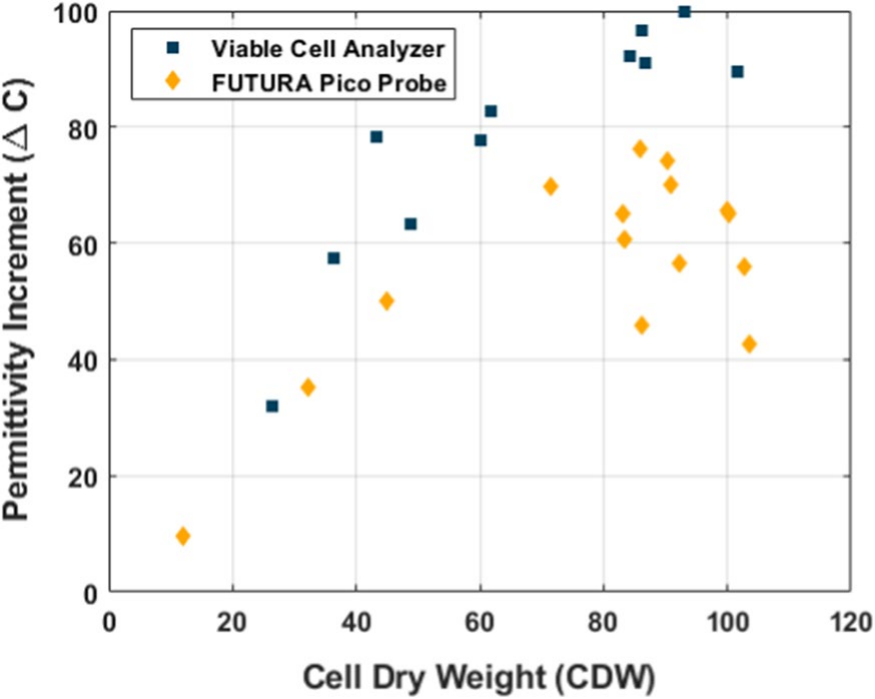
Comparison of measured CDW and ΔC. Dielectric spectroscopy of certain samples is measured in the Viable Cell Analyzer and others using the annular probe. No single sample is measured on both instruments

The classical issue as described in the introduction for a calibration method based only on measured Cell Dry Weight (CDW) is observed also for this system (Fig. 1): At the lower biomass concentrations in the early phase of the process, there is a strong linear relationship between CDW (g/kg) and Permittivity Increment ($\Delta C)$. At higher biomass concentrations, corresponding to middle and late phases, this correlation weakens significantly. It is suspected that the major deviations observed here are due to precipitations of insoluble fermentation products, which do not affect dielectric spectroscopy results. This is further illustrating that the total CDW measurement is a poor estimate of viable biomass. This is in agreement with older monitoring studies on filamentous fungi systems that dielectric spectroscopy should not fail at high biomass concentrations or with morphological changes (Krairak et al. 1999).

**Fig. 2:**
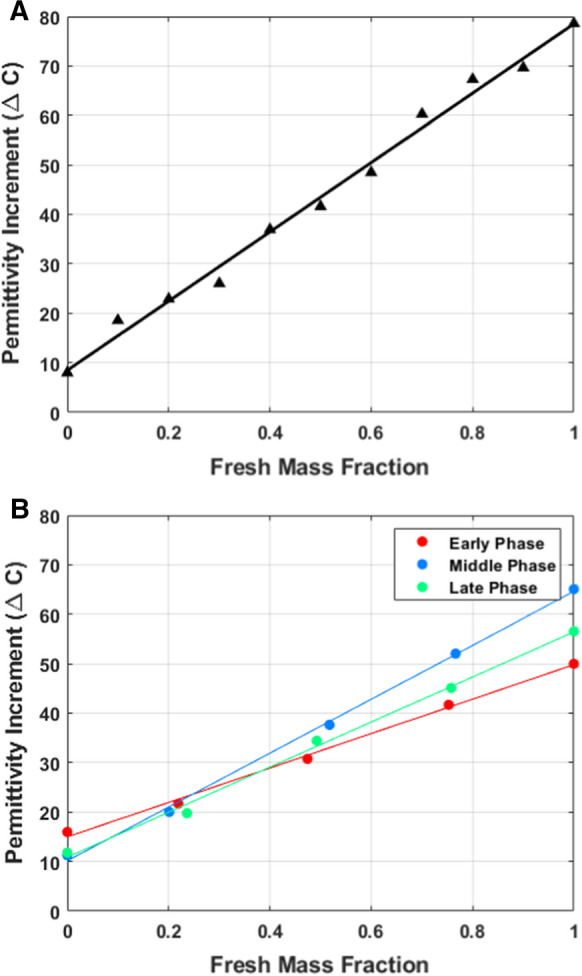
Differences in permittivity increment (ΔC) at different dilution levels. Figure 2a Shows a single sample in the stationary phase measured on the Viable Cell Analyzer at an increment of approx. 0.1 fresh sample mass fraction. Figure 2b shows the permittivity increment of three selected samples to represent different fermentation phases at different dilution levels, measured on the Annular Probe

The results for mixed killed/fresh samples demonstrate the anticipated linear response to the mass fraction of fresh vs. killed sample: One sample was measured at 10% intervals of the fresh mass fraction to verify linearity Fig 2a), samples selected so that each phase of the fermentation is represented (early, middle and decline) are also linear (Fig. 2b). These linear results are expected when $\Delta C$ is linearly correlated with viable biomass concentration. The results confirm the expected linear relationship between permittivity (reported as ∆C pF/cm) and viable biomass concentration for the entire fermentation duration.

### Dielectric spectroscopy calibration to viable biomass concentration

**Fig. 3:**
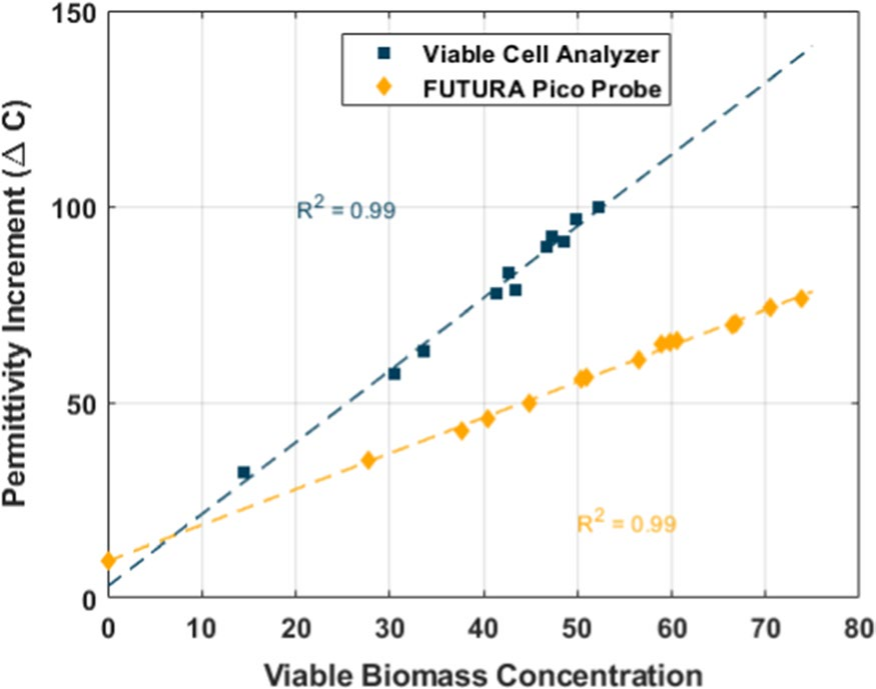
Determination of hidden relation between viable biomass and ΔC after applying the viable fraction corrections on CDW measurements. Two independent calibrations are obtained depending on the measurement device used for ΔC

Following the calibration methodology, for both legacy and modern instruments, values for $\mathrm{\alpha }$ were found for each of the analyzed samples that gave an extremely strong linear correlation between ∆C pF/cm, and the implied viable biomass concentration g/kg (Fig. 3).

While α was constrained to values less than or equal to 1, it is important to consider that the vector $\mathrm{\alpha }$ containing the viability fractions, can be scaled with a single scalar without a drop in ${R}^{2}$ value.This is equivalent to assuming that at least one of the measured samples may consist of only viable biomass as the solid phase. This may not always be a realistic assumption. However, as a correction to CDW, only non-active biomass is subtracted from the measurement and is thus a better approximation to active biomass than the original CDW measurement while at the same time being a much faster measurement technique, requires lower sample volumes and with modern probes, can be used for *online* data collection.

Linear calibration of β_1_ & β_2_ is displayed in Table 1. The probe generally measures lower $\Delta C$ than the legacy viable cell analyzer for similar viable biomass concentrations and strain. The difference can be explained because the probe and instrument utilized different measurement techniques at different frequencies. The important thing to note is that the signal is still able to establish a linear trend between ΔC and viable biomass, each method can be calibrated easily. These calibrations can be utilized directly to measure viable biomass concentration using dielectric spectroscopy in the current fermentation system.

**Table 1:** Parameters for the linear calibration between $\Delta C$ and viable biomass across the three different measurement types

Measurement instrument	Slope (${\upbeta }_{1}$)	Intercept (${\upbeta }_{2}$)
Viable Cell Analyzer	$1.67 \pm 0.05$	$9.49 \pm 1.39$
Futara Pico probe (2 mL)	$0.89 \pm 0.02$	$11.91\pm 0.76$
Futara Pico (100 mL)	$1.18 \pm 0.12$	$15.84\pm 0.83$

### Sample volume required with modern probes

All fresh samples measured with the modern probe were also measured in a larger sample container to examine the influence of the container wall effect on such small sample volumes. As expected, there was a difference in the $\Delta C$ reading depending on the container size due to close proximities between the electrode and container wall in the smaller tubes. However, the $\Delta C$ difference between large and small samples is very consistent throughout, thus the wall effect is largely the same as long as the container volume is kept consistent throughout a sampling campaign. A small sample of 2 mL can be used for direct *offline* measurements, but the scale difference must be noted if one intends to utilize the same calibration between different sample volumes or when transitioning to *on-line or in-line* monitoring.

**Fig. 4:**
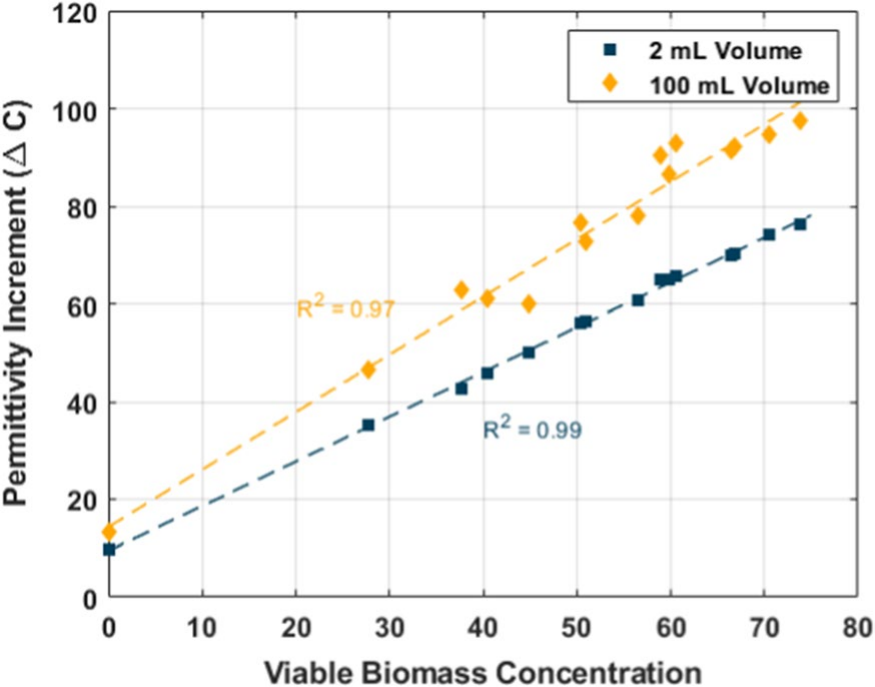
The difference in ΔC when measuring the same fresh samples with different sample volumes when using the annular probe. Viable biomass is calculated using the linear correlation calibrated with the 2 mL samples measured with the annular probe

Figure 4 shows the comparison between when the same fresh samples are analysed using the probe in a small tube container of 2 mL and a larger container of 100 mL, which is large enough to achieve a better resolution of the permittivity signal without artificial shifts. The viable biomass depicted in Fig. 4 is calculated using the linear constants from Table 1 with the 2 mL samples and directly compared to the $\Delta C$ measured on 100 mL samples. A new linear relation was constructed which is also illustrated in Fig. 4 utilizing the 100 mL samples and the constants are shown in Table 1. It was established by (Fernandes et al. 2019) that moving from *offline* to *online* could be done with a simple scalar conversion factor. This scaling factor is likely dependent on the microbe and measurement techniques and thus has to be determined for a specific system. While other studies concluded that *offline* analysis could be done with a modern probe as long as the electrode was not close to a surface to measure the full signal, the results here establish that it is possible to go even further down in the sample volume while retaining the information of interest. The conversion factor for this microbial system when estimating $\Delta C$ of a 100 mL sample utilizing 2 mL measurement was estimated to be 1.33 ± 0.07. A similar method of a scaling factor could be utilized for 2 mL *offline* samples to calibrate the probe for *online* monitoring.

## Conclusions

This work explored the calibration of dielectric spectroscopy measurements to viable biomass concentrations in an industrial fermentation system utilizing a filamentous fungus, using off-line measurements of various sample volumes, with legacy and modern equipment. Exercising some viability control, where a fresh sample containing an unknown fraction of living biomass is mixed with a killed portion of the same sample, indicates a strong linear relationship between viable biomass and Permittivity Increment ($\Delta C$), in all cases, at every stage of the fermentation, while classically, the correlation of $\Delta C$ and Cell Dry Weight (CDW) did not exist past a certain fermentation phase. For the first time, a simple numerical calibration methodology was developed using $\Delta C$ measurement from the mixed fresh and heat-killed samples. This method provides better estimations of viable biomass concentration without going through the long and difficult procedure of calibration with independent viability measurements, such as CFU determinations (of dubious value for a filamentous organism) or fluorescent staining with quantitative image analysis. The constants for the linear relationship were specific to the instrument type, sample volume, and container diameter. The constants may be more generally applicable to other fermentation processes or organism types, exploring this would require additional work but which could be done in other laboratories where the results may also be useful. The present calibration is applied in modelling and scale down work underway in our laboratories and production facilities.

## Data Availability

The datasets generated during and/or analyzed during the current study are not publicly available due to industry-sensitive information but are available from the corresponding author on reasonable request.

## List of symbols

$\Delta C$: Permittivity increment in the radio frequency region
${\upbeta }_{1}$: Slope of linear calibration
${\upbeta }_{2}$: Linear calibration offset
${X}_{Viable}$: Viable biomass concentration
${X}_{Dry}$: Total dry weight concentration
$\mathrm{\alpha }$: Dry weight viability fraction
${\mathrm{\alpha }}_{opt}$: Dry weight viability fraction estimated via optimization
${M}_{Frac}$: Mass fraction of non-heat treated broth in diluted samples
CDW: Cell Dry Weight (g/kg, filtered)
